# A nation-wide study on snus and smoked tobacco: The Swedish Tobacco Cohort (SWETOC)

**DOI:** 10.1177/14034948251350193

**Published:** 2025-06-25

**Authors:** Magnus Olsson, Eva Nordendahl, Björn Klinge, Michael Fored, Johan Sundström, Anders Ekbom, Hanne Tønnesen, Stefan Gustafsson, Aron Naimi-Akbar

**Affiliations:** 1 Faculty of Odontology, Health Technology Assessment-Odontology (HTA-O), Malmö University, Malmö, Sweden; 2 Clinical Epidemiology Division, Dept of Medicine, Karolinska Institutet, Solna, Sweden; 3 Dept of Periodontology, Eastman Institute, Folktandvården Stockholm AB, Stockholm, Sweden; 4 Periodontology and Dental Prophylaxis Unit, Dept of Dental Medicine, Karolinska Institutet, Huddinge, Sweden; 5 Dept of Medical Sciences, Clinical Epidemiology, Uppsala University, Uppsala, Sweden; 6 Karolinska Institutet, Karolinska University Hospital Solna, Department of Medicine, Stockholm, Sweden; 7 WHO-CC for Evidence-based Clinical Health Promotion, The Parker Institute, Bispebjerg-Frederiksberg Hospital, University of Copenhagen, Frederiksberg, Denmark

**Keywords:** Smoking, snus, snuff, cohort, Sweden, tobacco, nicotine, lifestyle, SWETOC, health

## Abstract

**Aims:**

Smoking increases the risk of developing severe diseases. However, the effects of snus are less known. We aimed to create a cohort on tobacco exposure by collecting data from the Swedish Public Dental Service. The cohort will be used to study even rare health outcomes of both smoking and snus.

**Methods:**

In Sweden, 35% (2021) of the adult population and 85% (2021) of all children use the Public Dental Service. The dental practitioner asks about tobacco use and registers replies in patients’ records. We have now assembled a database of all available such data in the country and linked them to other national health and social registers, forming the Swedish Tobacco Cohort (SWETOC). SWETOC is hence a cohort with prospectively designed data collection.

**Results:**

Out of all 21 regions in Sweden, 19 participated, and approximately 5.5 million unique individuals provided tobacco data. Registrations dated from 1994 to 2023. All participating regions provided data from at least 2015 and forward. Overall prevalence for smoking was 12%, and 13% for snus. More men used snus than women, and there were regional differences in tobacco use patterns. Gender distribution was around equal at all age levels. Some regions provided additional tobacco information such as amount and type of product used, willingness for tobacco cessation, and notes in free text.

**Conclusions:**

**SWETOC is a novel resource that can be used to close the current and future knowledge gaps regarding the health outcomes of smoked and smokeless tobacco.**

## Introduction

Globally, smoking accounts for approximately 6 million deaths annually, with the expectation of an increase to 8 million by 2030. There is overwhelming evidence that smoking tobacco increases the risk of cardiovascular disease, cancer, chronic respiratory disease, diabetes, and premature death [1, 2]. Even though the health effects of snus and other emerging forms of tobacco and nicotine products are less known, several studies suggest a negative impact on health, snus included. Studies on snus have found health effects on, for example, respiratory disease, cardiovascular disease, the health of the developing child [3], and increased risk of colorectal cancer [4]. One study has shown an increased overall mortality among prostate cancer patients [5]. Further, nicotine itself has a direct effect on blood pressure and has been linked to several health-related problems [6]. It is clear that snus affects health in several ways. In order to investigate this further, a large cohort like SWETOC could be very helpful, not least when investigating rare outcomes, where previous, less sizable cohorts have been unable to gain enough power [4, 7–9]. Smokeless tobacco is obtained from various commercial and homemade products. The common denominator, nicotine, is expected to have consistent dose-dependent health effects regardless of the formulation. The exposure and blood levels of nicotine in regular snus users are similar to those of regular cigarette smokers, although nicotine absorption from snus is prolonged and slower than that of smoking [10, 11].

In Sweden, approximately 5% of men and women aged 16–84 years are regular daily smokers, and 21% of men and 9% of women in the same age group use snus daily (2023) [1]. Snus is a smokeless tobacco product that is placed under the lip, allowing nicotine to be absorbed through the mucous membrane. While smoking in Sweden has been decreasing for decades, snus use has been increasing in men and women, especially among younger individuals [7]. Many new types of snus on the market have contributed to this [12]. Hence, the prevalence of tobacco use among men in Sweden is similar to that in most other European countries; however, smoking rates are relatively low as many men prefer to use snus. Swedish men had the lowest percentage of deaths related to tobacco use among developed countries. This has been claimed to be because snus is a less harmful alternative to cigarettes [13].

The concept of harm reduction involves substituting a more dangerous product with a less dangerous one to reduce total tobacco-related mortality and morbidity [14]. The smokeless tobacco industry promotes its products by reducing harm. However, general critics argue that no form of tobacco should be promoted, as it distracts from the overall goal of tobacco elimination [14]. Furthermore, snus may be important as a gateway to cigarette use [15].

This project was initiated in 2020 as an investigator-driven collaboration between Swedish researchers. With a high prevalence of snus use and strong registry infrastructure, Sweden holds unique possibilities for studying the health effects of snus use. Snus use and smoking have implications for other forms of smokeless tobacco products [16]. Answering these and other important questions regarding the risks associated with tobacco and nicotine use is imperative. Furthermore, the total impact of these products on healthcare use and cost remains unclear.

This research project aimed to examine the health effects of tobacco exposure by constructing a national dataset containing information on tobacco exposure based on information retrieved from the Swedish Public Dental Service [17]. Data are linked to national health registers, and the cohort is used to study the rare health outcomes of smoking and snus. The cohort will be updated, which adds to the follow-up time and provides information on newer products available.

This paper describes the SWETOC cohort, data collection process, collected data, harmonization, and results.

## Methods

### Study design

The SWETOC is a population-based cohort study of prospective registry data. Sweden has several registry resources. Together, governmental agencies, the National Board of Health and Welfare (SoS), and Statistics Sweden (SCB) host multiple important individual-level data for epidemiological research. Information from registries can be linked using a personal identity number (PIN) unique to all Swedish citizens [18].

### Study population

The study population include all who visited the Swedish Public Dental Service (Folktandvården) between 1994 and 2023 and have a Swedish PIN. The data gathered for this cohort included all records related to tobacco exposure in health declaration forms (Table I).

**Table I.** Characteristics of the total cohort, all ages, divided on sex and year born.

	Women		Men		Total	
	*n*	%	*n*	%	*n*	%
	28,00,201	50%	27,46,389	50%	55,46,590	100%
≤ 1940	2,26,793	8%	1,74,911	6%	4,01,704	7%
1941–1960	4,50,933	16%	4,38,175	16%	8,89,108	16%
1961–1980	6,66,984	24%	6,30,663	23%	12,97,647	23%
≥ 1981	14,55,491	52%	15,02,640	55%	29,58,131	53%

### Data collection

After obtaining approval from the Ethics Review Authority on 25 August 2020 (No: 2020-01700), data collection for the base cohort was initiated. All 21 regions in Sweden were contacted via email, followed by presentations of the project during online meetings. Documentation related to the project, including a research plan, ethical approval, and description of the desired data, along with specifications on the delivery of the data, were sent to the regions. All regions, excluding Jönköping and Gotland, agreed to participate.

**Figure 1.**
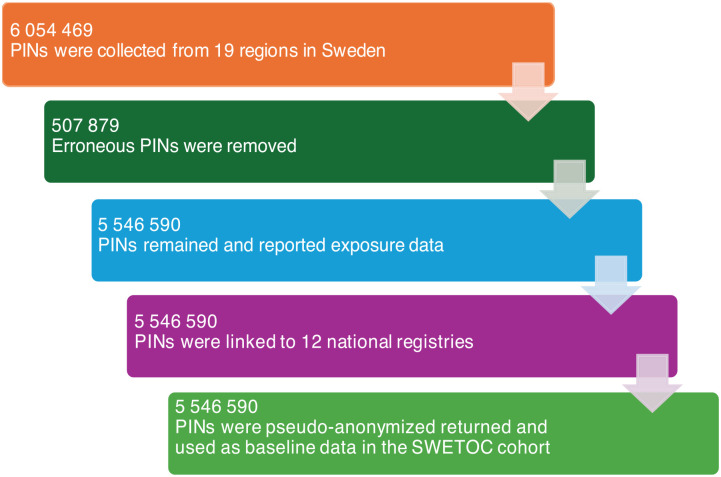
Simplified summary of the data collection and harmonization process, resulting in a dataset of 5,546,590 unique patients/PINs (personal identity numbers) reporting exposure data, and linked data from 12 national registers. Erroneous PINs were for example reused, temporary, or test PINs.

Exposure data, along with personal identity numbers and registration dates for each dental visit, were collected. All data were encrypted before being sent to Malmö University by registered post. Once retrieved, the exposure data were harmonized and stored on a secure server at Malmö University. The final dataset was sent to Statistics Sweden (SCB) for pseudo-anonymization and linkage to health registers (Figure 1).

### Data sources

#### Swedish Public Dental Services

Sweden is divided into 21 self-governing regions, and Swedish Public Dental Services are managed locally by these regions [19]. Regional dental clinics have their own electronic dental record systems. Although most regions have similar setups, this implies differences in data sources. Each dental record contains a general health and medication form. This form contains questions regarding tobacco use and is mandatory for dental practitioners to fill out and update. The dental practitioner interviews the patient at the clinic, following the form, and registers the answers in the dental record. The electronic system stores the data in a regional database. Thus, information regarding tobacco use is available at each dental visit and can provide longitudinal data on tobacco use.

Reasons for visiting the dental clinic vary from emergency visits to regular checkups, treatments, extractions, etc. Not all visits required an updated health form. Ages also vary but most children go to the Public Dental Service. Approximately 35% (2021) of adults and 85% (2021) of children and adolescents receive dental care through the Public Dental Service [1, 20]. There is also a private sector for dental care, and there might be a socioeconomic difference between the two. Regional differences exist in access to dental care and waiting times for appointments. Over the last 10 years, more individuals have moved from the public sector to the private sector. Older patients tend to move to the private sector over time [1, 21].

#### Health and social registers

**Figure 2.**
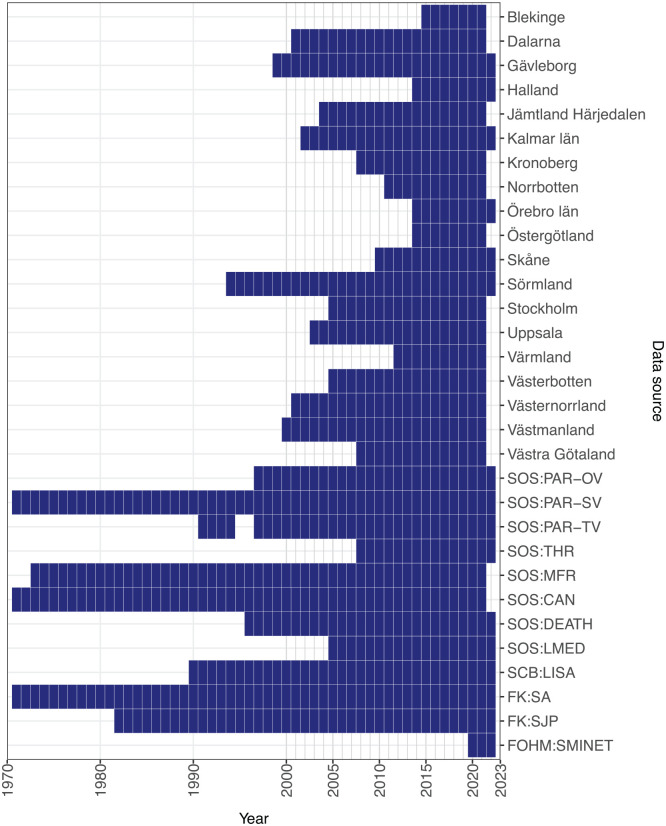
Overview of data sources and available years, and the 19 regions followed by linked registers.

Applications for linkage to health and social registers were submitted to the SoS, Swedish Social Insurance Agency (FK), Public Health Agency (FoHM), and SCB. This process resulted in 12 health and social registers (Figure 2).

All medical events that result in admission or visit to any hospital (diagnoses classified using the International Classification of Diseases (ICD) system) [22], a surgical procedure (classified using the Nordic Medico-Statistical Committee Classification of Surgical Procedures (NCSP) system), a filled drug prescription (classified using the Anatomical Therapeutic Chemical Classification (ATC) system), or death are collected systematically in the National Inpatient Register [23] and the Cause of Death Register [24], these registers having virtually full coverage in Sweden since 1987 and 1961, respectively [18]. The high level of detail in these registers enables the possibility of studying the associations among exposure, outcome, and comorbidity. Furthermore, important background data, such as heritability and socioeconomic circumstances, are available in the multi-generation registry [25] and longitudinal integrated database for health insurance and labour market studies (LISA) [26]. Even more detailed information about sick leave in Sweden is provided by the Sweden Micro-Data for Analysis of the Social Insurance System (MiDAS) database, maintained by the Swedish Social Insurance Agency. (A complete list of registers that have approved linkage can be found in Table S1).

### Ethical considerations

This study was approved by the Ethics Review Authority and all participating registry holders. The project involved collecting personal data linked to national registers. Ethical problems regarding the participants’ integrity are limited because the data are pseudo-anonymized before they are linked to registries. Depending on the size of the dataset and because data will be handled in agreement with the GDPR, the risk of identifying individuals is low. Overall, the possible benefits for future users of tobacco and nicotine obtained from this project seem considerably higher than the low risks of identification and data leakage.

### Material and software

Raw exposure data were processed at Malmö University. Further harmonization and data analysis were enabled by resources in project sens2022573 provided by the National Academic Infrastructure for Supercomputing in Sweden (NAISS) at UPPMAX, funded by the Swedish Research Council through grant agreement no. 2022-06725. For all statistical analysis, *R* version 4.3.1 (2023-06-16) and *R*-Studio were used [27, 28]. Power analyses were conducted for rare outcomes comparing snus users (*n* = 510,303) to non-tobacco users (*n* = 3,722,775), based on publicly available data on incidence, from the National Board of Health and Welfare [29]. Incidence rates for men and women 20 years and older were collected. Follow-up time was assumed to be eight years. The incidence rate for oral cancer was 10.9, oesophageal cancer 6.5, and pancreatic cancer 17.3 per 100,000 person years. We included analyses for the following hazard ratios (HRs): 1.05, 1.15, 1.25. The number of required events, expected events, and power was calculated. For sample size calculations, a power of 0.80 and significance level of 0.05 were used.

### Results

Out of the 21 regions, 19 participated and reported registry data. Approximately 6 million patients were extracted from the regional databases and sent for linking to the national databases (Figure 1). After the removal of reused and errant PINs, approximately 5.5 million patients remained. All regions provided data on patients younger than 18 years old and 1.9 million patients occur at least once in this age group. In most studies, adults will be of greater interest. Descriptive statistics in this report, except for those shown in Table I, are therefore based on people aged 18 to 110 years old. This group contributed to 19.4 million dental visits. All regions reported data from at least 2015. The overall prevalence of smoked tobacco was 11% and snus was approximately 12% (Tables IIa and IIb, and Figure S3). Men use more snus than women, and there are regional differences in tobacco use. The sex distribution was equal at all age levels (Table I). Some regions reported additional information, such as the amount and type of tobacco used, smoking cessation, and notes, in the free text (Table S2). A simple validation of exposure data was done by calculating the incidence of lung cancer estimated in a Kaplan–Meier analysis, stratified by age group and smoking status among smokers in the cohort and comparing the result to previous studies (Figure S1) [30]. Another simple validation of linked registers was done by visualizing the proportion of individuals who had an inpatient visit with the principal diagnosis from a given ICD10 chapter (A–Z) (Figure S4). The annual median income was extracted from linked data, and was approximately 344 100 SEK (2019, ages 18–64). To show the differences in snus exposure between regions, a map was plotted for the year 2019 (Figure S2).

**Table IIa.** Summary statistics showing number of visits, patients, smokers, snuffers, percent of smokers, and percent of snuffers per region. Ages 18–110 years old. The summary is based on all visits and years.

Region	Visits (*n*)	Smoker (*n*)	Smoker (%)	Snuffer (*n*)	Snuffer (%)	Both (*n*)	Both (%)
Blekinge	148,031	21,297	14%	18,149	13%	3,173	2%
Dalarna	790,621	89,619	12%	103,227	14%	11,893	2%
Gävleborg	1,311,702	187,005	14%	196,133	15%	37,993	3%
Halland	227,053	24,166	12%	32,064	16%	3,066	2%
Jämt. Härjedalen	312,609	27,814	12%	50,332	20%	4,234	2%
Kalmar län	677,466	80,331	13%	86,000	14%	12,703	2%
Kronoberg	473,143	54,824	13%	53,208	13%	3,790	1%
Norrbotten	324,393	27,364	8%	49,407	15%	4,727	1%
Örebro län	482,247	58,240	12%	62,319	13%	9,039	2%
Östergötland	381,074	49,593	13%	45,643	13%	6,906	2%
Skåne	2,055,013	166,723	8%	158,617	8%	17,177	1%
Sörmland	831,329	129,089	16%	78,660	11%	12,091	2%
Stockholm	4,048,488	464,263	12%	397,631	10%	59,239	2%
Uppsala	723,266	57,932	9%	67,845	10%	7,997	1%
Värmland	398,739	31,487	8%	52,910	13%	3,924	1%
Västerbotten	893,721	78,116	10%	142,550	17%	11,654	2%
Västernorrland	727,853	80,818	12%	101,972	15%	11,931	2%
Västmanland	777,401	94,576	14%	92,229	14%	16,678	3%
Västra Götaland	3,870,346	315,529	8%	412,782	11%	43,793	1%
Total	19,454,495	2,038,786	11%	2,201,678	12%	282,008	2%

**Table IIb.** Summary statistics showing number of visits and patients per region. Ages 18–110 years old. The summary is based on all visits and years.

Region	Visits (*n*)	Patients (*n*)
Blekinge	148,031	51,053
Dalarna	790,621	186,802
Gävleborg	1,311,702	237,873
Halland	227,053	93,215
Jämt. Härjedalen	312,609	80,470
Kalmar län	677,466	157,116
Kronoberg	473,143	106,088
Norrbotten	324,393	122,918
Örebro län	482,247	139,737
Östergötland	381,074	108,374
Skåne	2,055,013	530,492
Sörmland	831,329	259,068
Stockholm	4,048,488	1,057,223
Uppsala	723,266	210,215
Värmland	398,739	126,062
Västerbotten	893,721	204,439
Västernorrland	727,853	165,956
Västmanland	777,401	168,112
Västra Götaland	3,870,346	852,107
Total	19,454,495	4,529,287

### Collected data

All regions reported data on smoking and snus. Additionally, some regions reported the amounts used. The unit varies among and within regions. In some regions, the amount was reported in free text, whereas other regions had a preselection of choices for the practitioner. Some regions reported the time spent as tobacco users, smokers, snuffers, and both. Some regions have dedicated fields for free text input. In this field, information such as the amount, frequency, tobacco products used, and willingness to quit is recorded (Table S2).

## Discussion

In this novel nation-wide resource with tobacco exposure based on data collected from the Swedish Public Dental Service, 19 out of 21 healthcare regions are represented, with data from approximately 5.5 million unique patients between 1994 and 2023. This resource provides aspects of repeated self-reported information, allowing longitudinal examinations of tobacco use patterns and linkages to national registry data, facilitating investigations of the associations between tobacco use patterns and health and societal outcomes.

To study the health effects of snus is important due to the increase of snus use, especially among young people, where risks are currently unknown. This cohort will be able to provide new insights and knowledge regarding risks with snus, over shorter and longer periods. Previous studies have investigated the relationship between snus and cancer. For example, two larger studies on pancreatic cancer and snus showed varying results. Luo et al. (2007) showed an increased risk among snus users (OR – 2.0) and Araghi et al. 2017 showed no such association (HR – 0.96) [9, 31]. SWETOC will likely contribute to this field of research and help to fill these important gaps in the scientific evidence with the large sample size and valuable exposure data.

### Strengths and limitations

One of the greatest strengths with this cohort is the size, which gives the ability to investigate even rare outcomes with sufficient statistical power, which has been a concern in similar previous studies [4, 8]. Since tobacco use was self-reported, there was a risk of reporting bias because of the possibility of underestimation of tobacco use. On the other hand, it is a strength that exposure is very seldom overestimated. In addition, patients were interviewed by health professionals at the clinic, which in previous studies has shown a higher data quality and less misreporting compared to questionnaires [32]. Due to the open cohort design based on data from medical records, the inclusion rate was very high, and dropout was not a problem, except that some patients may move to the private sector over time and therefore be lost to follow-up over time. The open cohort further allows for continuous data collection and regular linkage to national health and social registers. It is also a strength that all harmonization and data development are processed at the same supercomputer cluster UPPMAX at Uppsala University, which can handle sensitive personal information.

In all the steps of data collection, there is a risk of human error and non-differential misclassification bias, e.g., older documentation of smoking would remain in the records until the next follow-up although the patient might have changed habits. Furthermore, tobacco-free products, such as nicotine pouches or e-cigarettes, could have been reported as tobacco, which is a form of observer bias, and systematic errors might skew the data if a region decides to report smokeless tobacco as cigarettes (an unknown error in the data). Random errors can lower the precision. Typographical errors could occur in regions in which the amount is reported as free text. There might be a risk that those going to the Public Dental Service are not completely comparable to the general population in Sweden, and this might affect the external validity of our cohort. However, when comparing point estimates on demographics such as sex and socioeconomic factors such as median income per year to publicly available data, the present study seems to be aligned with publicly available statistics for the Swedish population, suggesting representativity [21, 33]. Similarly, the exposure data seems valid, as time to lung cancer among smokers in the present study shows similar results compared to previous studies (Figure S1) [30]. To further address the potential risk of selection bias, we plan to apply inverse probability weighting using publicly available sociodemographic data. This approach will allow us to account for differences between our study population and the general population. The resulting weights will be incorporated into the cohort data to improve representativeness and ensure more accurate and generalizable estimates in future analyses.

### Perspectives

When utilized in future studies, the SWETOC cohort may contribute with new knowledge on the risks of smokeless as well as smoked tobacco, knowledge that could help patients make informed choices on tobacco use and support them to quit successfully. New knowledge could also be used for preventive measures and intervention for the benefit of the society at large, saving lives and money. Future research would benefit from standardized documentation of tobacco and nicotine in medical records across regions and sectors, public as well as private.

### Significance

Utilizing healthcare data for research is a key goal in the Swedish national life science strategy. The Swedish Public Dental Service is a nation-wide coherent organization that regularly follows individuals in dental care. Here, we describe a novel resource utilizing dental care data for healthcare research. By constructing a national dataset with repeated, structured collection of self-reported data on tobacco exposure, both smoking and snus, we enable detailed study of even rare health effects of tobacco and help decision-makers save lives and money.

## Conclusion

SWETOC is a novel resource that can be used to close the current and future knowledge gaps regarding the health outcomes of smoked and smokeless tobacco.

## Supplemental Material

sj-docx-1-sjp-10.1177_14034948251350193 – Supplemental material for A nation-wide study on snus and smoked tobacco: The Swedish Tobacco Cohort (SWETOC) Supplemental material, sj-docx-1-sjp-10.1177_14034948251350193 for A nation-wide study on snus and smoked tobacco: The Swedish Tobacco Cohort (SWETOC) by Magnus Olsson, Eva Nordendahl, Björn Klinge, Michael Fored, Johan Sundström, Anders Ekbom, Hanne Tønnesen, Stefan Gustafsson and Aron Naimi-Akbar in Scandinavian Journal of Public Health
