# Supplementary material for: A nation-wide study on snus and smoked tobacco: The Swedish Tobacco Cohort (SWETOC)
Source: Scand J Public Health. 2025 Jun 25;54(6):672–9. doi: 10.1177/14034948251350193 (PMC13356259; doi:10.1177/14034948251350193)
Supplement: sj-docx-1-sjp-10.1177_14034948251350193 – Supplemental material for A nation-wide study on snus and smoked tobacco: The Swedish Tobacco Cohort (SWETOC) [file sj-docx-1-sjp-10.1177_14034948251350193.docx]

**Supplementary material - The Swedish Tobacco Cohort (SWETOC)**

**Table S1.** Health and social registries approved for linkage.

| **Registry holder** | **Name / description** | **Abbreviation** |
| --- | --- | --- |
| National Board of Health and Welfare | National Patient Register, outpatient care | PAR-OC |
|  | National Patient Register, inpatient care | PAR-IN |
|  | National Patient Register, compulsory care | PAR-TV |
|  | National Dental Health Register | DHR |
|  | Medical Birth Register | MBR |
|  | Swedish Cancer Register | CAN |
|  | Cause of Death Register | CDR |
|  | Prescribed Drug Register | PDR |
| Statistics Sweden | Longitudinal integration database for health insurance and labour market studies | LISA |
| Swedish Social Insurance  Agency | Microdata for Analyses of Social Security database, sickness benefit etc. | MiDAS-SA |
|  | Microdata for Analyses of Social Security database, early retirement etc. | MiDAS-SJ |
| Public Health Agency | Communicable disease surveillance register | SmiNet |

**Table S2.** Summary of reported years and variables of exposure data per region.

Green indicates that the variable exists.

| **Region** | **From** | **To** | **Free text** | **Amount** | **Cessation** | **Smoking variables** | **Snus variables** |
| --- | --- | --- | --- | --- | --- | --- | --- |
| Blekinge | 2015 | 2021 |  |  |  | Smoker, never, passive, recent | Snuffer, never, recent |
| Dalarna | 2001 | 2021 |  |  |  | Smoker daily | Snuffer daily |
| Gävleborg | 1999 | 2022 |  |  |  | Smoker | Snuffer |
| Halland | 2014 | 2022 |  |  |  | Smoker, never, daily, recent | Snuffer, never, daily, recent |
| Jämtland Härjedalen | 2004 | 2021 |  |  |  | Smoker, both, no smoking, | Snuffer, both, no snuff |
| Kalmar län | 2002 | 2022 |  |  |  | Smoker, smoker or snuffer | Snuffer, snuffer or smoker |
| Kronoberg | 2008 | 2021 |  |  |  | Cigarettes, pipe or waterpipe | Snuffer |
| Norrbotten | 2011 | 2021 |  |  |  | Smoker | Snuffer |
| Örebro län | 2014 | 2022 |  |  |  | Smoker, quit smoking | Snuffer, snuffer or smoker |
| Östergötland | 2014 | 2021 |  |  |  | Smoker, habits, package years, quit | Snuffer, habits, consumption |
| Skåne | 2010 | 2023 |  |  |  | Smoker | Snuffer |
| Sörmland | 1994 | 2023 |  |  |  | Smoker, habits, package years, quit | Snuffer, habits, consumption |
| Stockholm | 2005 | 2021 |  |  |  | Smoker, smoker and/or snuffer | Snuffer, smoker and/or snuffer |
| Uppsala | 2003 | 2021 |  |  |  | Smoker | Snuffer |
| Värmland | 2012 | 2021 |  |  |  | Smoker | Snuffer |
| Västerbotten | 2005 | 2021 |  |  |  | Cigarettes, cigars, habits | Snuffer |
| Västernorrland | 2003 | 2020 |  |  |  | Cigarettes, cigars, pipe, waterpipe | Snuffer |
| Västmanland | 2000 | 2021 |  |  |  | Free text only | Free text only |
| Västra Götaland | 2008 | 2020 |  |  |  | Cigarettes, cigars, pipe, waterpipe | Snuffer |

**Table S3.** Power analyses were conducted for rare outcomes comparing snus users

(n = 510,303) to non-tobacco users (n = 3,722,775), using incidence rates per 100,000 person-years for men and women aged 20 years and older, based on publicly available data. The follow-up period was eight years. Hazard ratios (HRs) of 1.05, 1.15, and 1.25 were examined. The number of required and expected events, as well as statistical power, were calculated. A power of 0.80 and a significance level of 0.05 were used.

| **HR** | **CANCER** | **INCIDENCE** | **EXPECTED_EVENTS** | **REQUIRED_EVENTS** | **POWER** |
| --- | --- | --- | --- | --- | --- |
| 1.05 | Pancreas | 0.000173 | 5859 | 31100 | 0.2284 |
| 1.15 | Pancreas | 0.000173 | 5859 | 3790 | 0.9361 |
| 1.25 | Pancreas | 0.000173 | 5859 | 1487 | 0.9998 |
| 1.05 | Oral | 0.000109 | 3691 | 31100 | 0.1599 |
| 1.15 | Oral | 0.000109 | 3691 | 3790 | 0.7895 |
| 1.25 | Oral | 0.000109 | 3691 | 1487 | 0.9929 |
| 1.05 | Oesophageal | 0.000065 | 2201 | 31100 | 0.1123 |
| 1.15 | Oesophageal | 0.000065 | 2201 | 3790 | 0.5695 |
| 1.25 | Oesophageal | 0.000065 | 2201 | 1487 | 0.9263 |


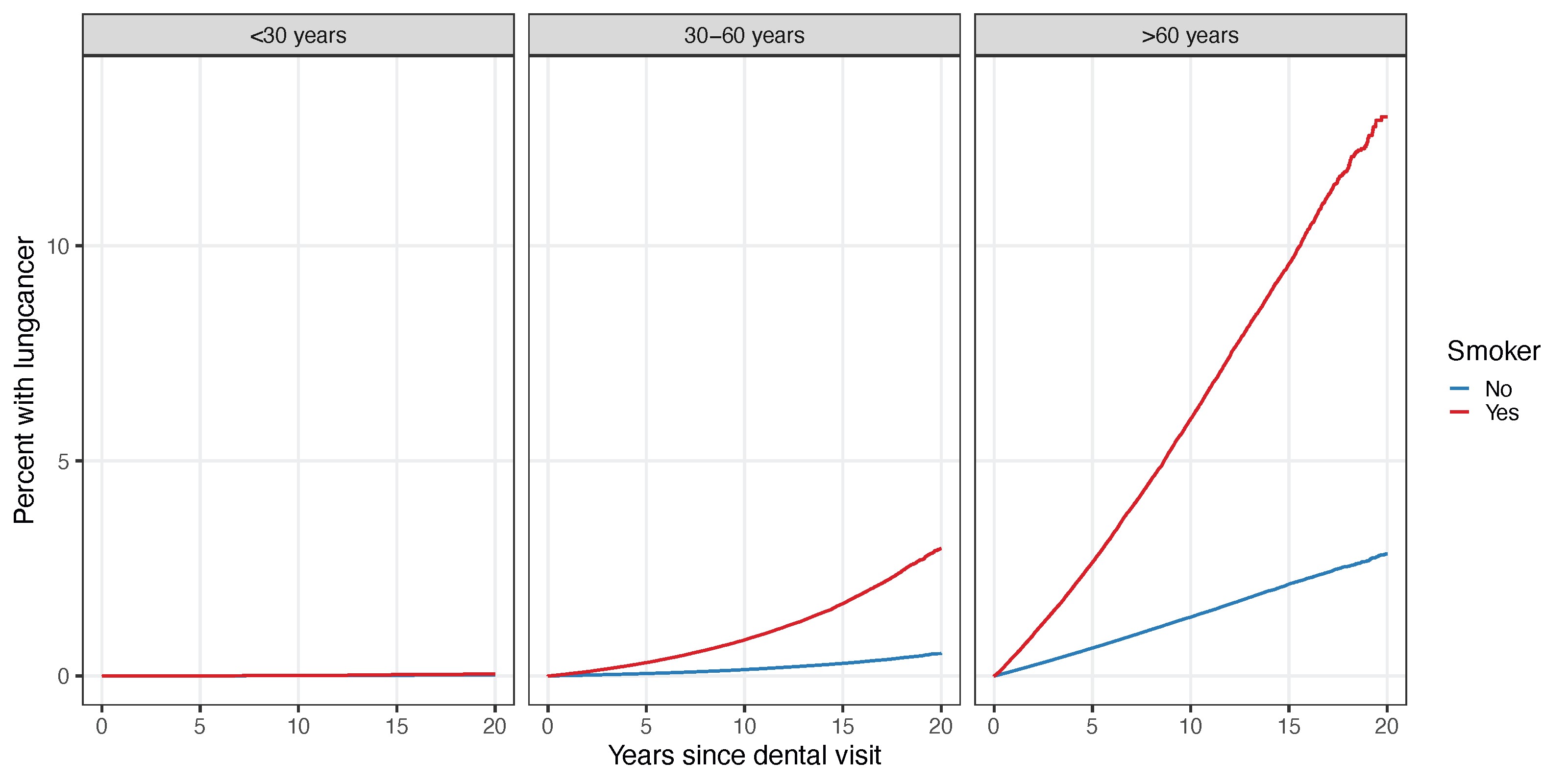


**Figure S1.** Incidence of lung cancer in patients free from lung cancer at time of dental visit (time=0) and followed for up to 20 years. The proportion with lung cancer estimated in a Kaplan-Meier analysis, stratified by age group and smoking status.


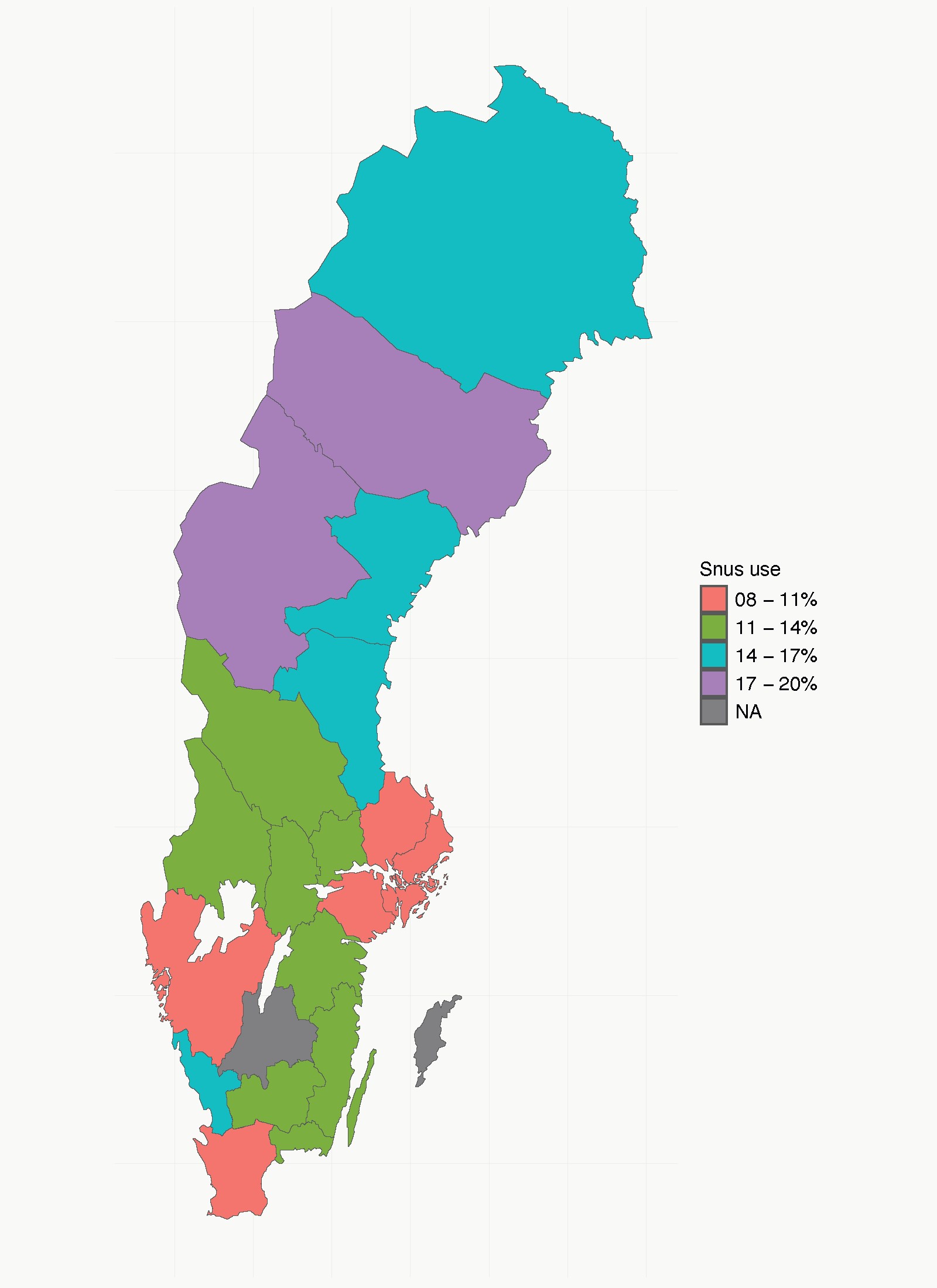


**Figure S2.** Map over Sweden with the regional distribution of snuffers for year 2019.


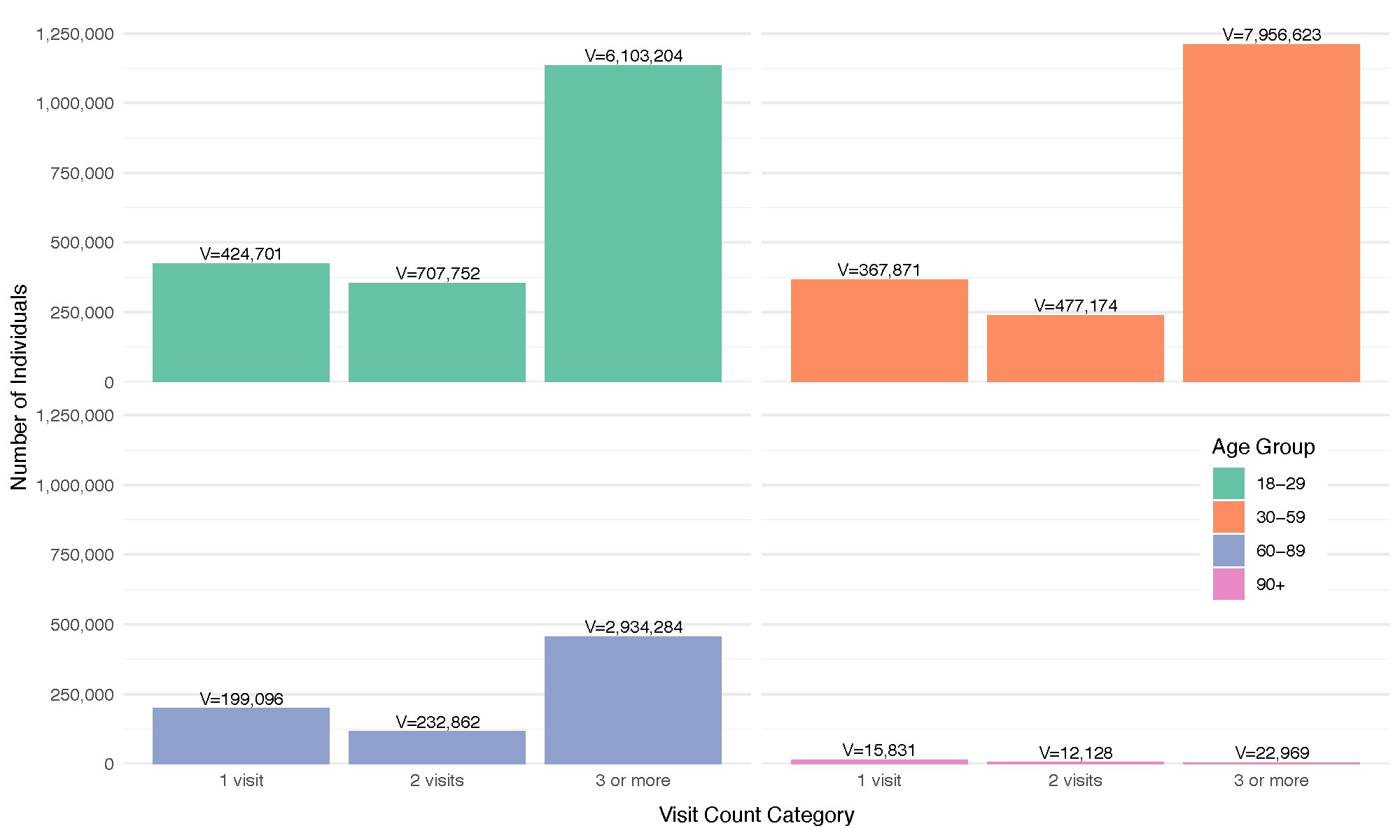


**Figure S3.** Summary statistics on visits and number of individuals, divided into four age groups and tree visit categories. V=the number of total visits inside that specific visit category.


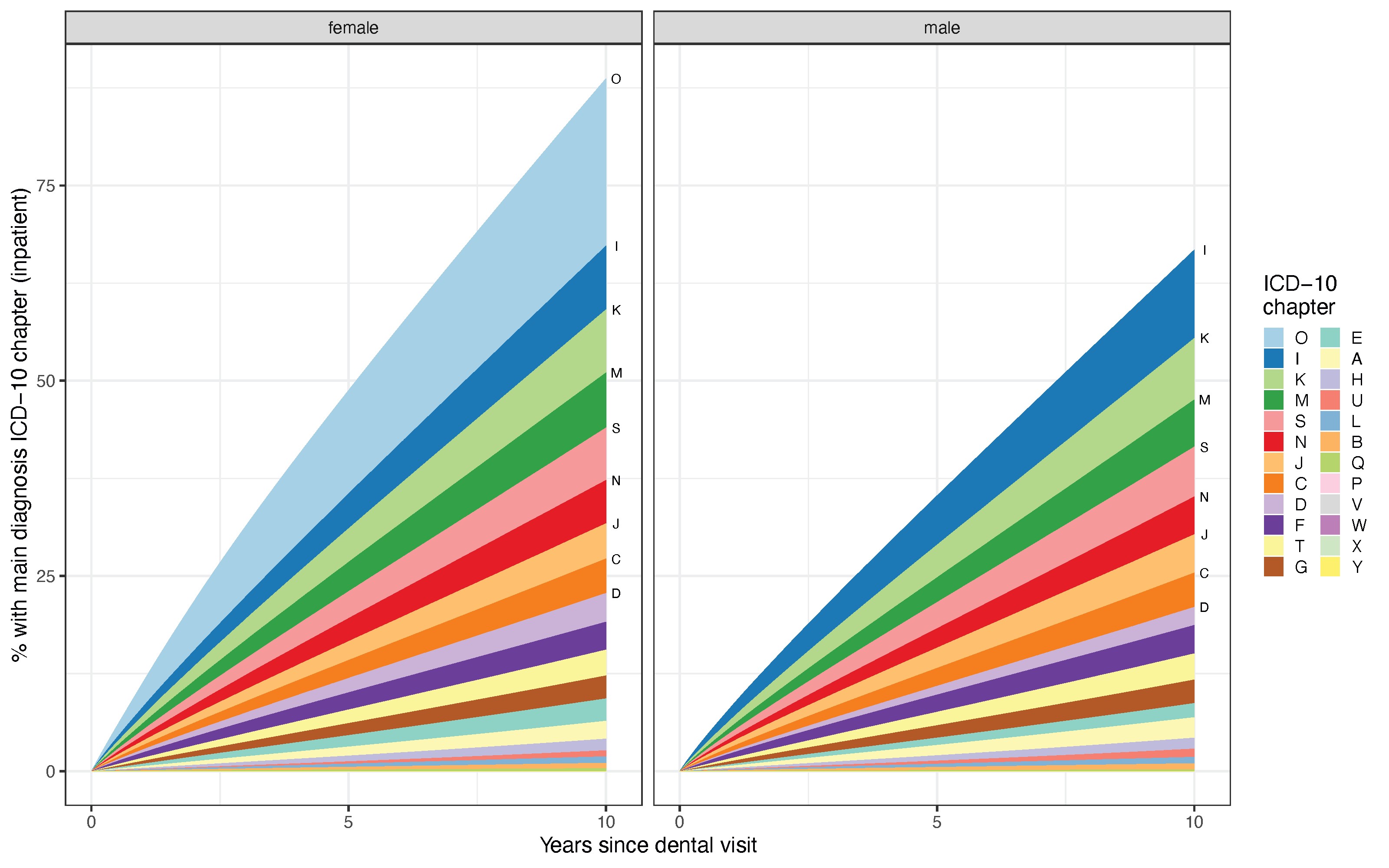


**Figure S4.** Proportion of individuals who had an inpatient visit with the principal diagnosis from a given ICD10 chapter (A-Z). Showing the % who received a given final care diagnosis (y-axis) over time (x-axis). Separate for men and women (mainly due to chapter O, pregnancy-related). Short form ICD-10 Chapters; A: Infectious diseases, B: Infectious diseases, C: Neoplasms (cancers), D: Blood and immune disorders, E: Endocrine and metabolic disorders, F: Mental and behavioural disorders, G: Nervous system, H: Eye and ear diseases, I: Circulatory system, J: Respiratory system, K: Digestive system, L: Skin disorders, M: Musculoskeletal system, N: Genitourinary system, O: Pregnancy and childbirth, P: Perinatal conditions, Q: Congenital malformations, R: Symptoms and abnormal findings, S:

Injuries and poisoning, T: Injuries and poisoning, U: Provisional/emerging diseases, V: External causes of morbidity, W: External causes of morbidity, X: External causes of morbidity, Y: External causes of morbidity, Z: Factors influencing health status.
